# Towards the Development of Microbial Ecotoxicology Testing Using Chlorpyrifos Contaminated Sediments and Marine Yeast Isolates as a Model

**DOI:** 10.3390/microorganisms10102019

**Published:** 2022-10-13

**Authors:** Gustavo Echeverri-Jaramillo, Beatriz Jaramillo-Colorado, Howard Junca, Claudia Consuegra-Mayor

**Affiliations:** 1 Grupo GIMA, Universidad de San Buenaventura, Cartagena 130010, Colombia; 2 Grupo GIA, Universidad de Cartagena, Cartagena 130027, Colombia; 3 Microbiomas Foundation, LT11A, Chía 280004, Colombia

**Keywords:** Chlorpyrifos, TCP metabolite, pollution, marine yeasts, microbial ecotoxicology, microbiological techniques

## Abstract

Chlorpyrifos (CP), a widely used pesticide, and its metabolite 3,5,6-trichloro-2-pyridinol (3,5,6-TCP), are xenobiotic compounds detected in many biomes, notably in marine sediments, all over the world. These compounds are posing a serious environmental and health problem given their toxicity to wildlife and possible exposure effects to human neurodevelopment. Microorganisms at CP-impacted environments could harbor metabolic capabilities that can be used as indicators of the biological effects of the contaminant and could encode selected functions reactive against contaminants. Those features could be used for microbial ecotoxicology applications by collectively using analytical, enzymatic, microbiological and toxicological techniques in order to assess the biological effects of pollutants and other environmental/climatic stressors in ecosystems. The objective of this study was to assess the variability in the metabolic responses of yeast isolates from CP-contaminated marine sediments as potential biological indicators for microbial ecotoxicology testing. Sediment samples from a South Caribbean tropical shore (Cartagena Bay, Colombia) were collected, and deoxyribonucleic acid (DNA) was recovered from lyophilized aliquots. The DGGE (Denaturing Gradient Gel Electrophoresis) technique targeting fungal Internal Transcribed Spacer (ITS) showed the great diversity of fungal types. Simultaneously, yeast strains were isolated from the freshly collected sediment samples. Physiological characterization including API 20C and antibiosis tests, growth patterns at salt concentrations (2/4/10/25%), temperatures (4/25/37/45 °C), esterase activity assay and resistance tests to CP/TCP toxicity resulted in 10 isolated yeast strains, identified as *Candida* spp. (6), *Cryptococcus* spp. (3). and *Rhodotorula* spp. (1), showing promising characteristics to be used as a test for yeast-based ecotoxicity indicators. The patterns of carbohydrate assimilation, low antibiosis, presence of esterases/lipases, growth in a wide range of temperatures and salt concentrations, and tolerance to minimal inhibitory concentrations of CP and TCP are factors useful for testing environmental samples.

## 1. Introduction

Chemical contamination by pesticides is an enormous environmental problem; coastal aquatic ecosystems, such as estuaries nearby large urban and industrial centers, are at a higher risk of impact and pollution [1]. Organophosphorus insecticides (OPI) such as Chlorpyrifos (CP), and its more water-soluble and persistent metabolite TCP (3,5,6-trichloro-2-pyridinol) [2,3], accumulates in waters, soils and sediments, affecting a large number of non-target organisms [4].

In natural aquatic environments, microorganisms interact with biotic and abiotic systems playing key processes in ecosystem functioning for food chain preservation, biogeochemical cycling, and biodegradation of contaminants. From this large microbial diversity, some microorganisms have evolved traits that can potentially be used for bioremediation and for microbial ecotoxicology testing [5]. Given the abundance and metabolic activity of environmental microbial communities, any alteration given by contamination affects the resistance of ecosystems [6,7,8,9,10,11]. Microbial ecotoxicology approaches are strengthened with the use of analytical, enzymatic, toxicological, and microbial culture methods, in order to have more precise evaluations regarding toxic pollutants in an ecosystem [6,12,13]. Traditional analytical methods have been used to confirm the degradation of contaminants (HPLC, GC-MS, ICPMS, etc.), but the short- and long-term toxicity to various organisms of the accumulated contaminants, traces or metabolites need to be additionally evaluated to have a broader assessment of their impacts. Methods reported in the literature consist of tests using individual species, measurements of carbon and nitrogen transformation, enzymatic tests, biomass measurements and changes in microbial diversity, among others [14,15]. Molecular techniques such as DGGE, Terminal restriction fragment length polymorphism (TRFLP) and next generation sequencing allow the culture independent detection of microbes present in a given sample, and a more precise detection of the complex composition in communities, of the microorganisms involved in the degradation of contaminants or the production of a specific enzyme [5].

Microbial ecotoxicology approaches use microorganisms isolated from different contaminated habitats to assess the effect of a chemical, which can be used as an indicator of a toxic effect [16]. The search for microorganisms in less accessible/explored environments such as marine ecosystems is promising as they are adapted to factors such as salt concentrations, temperatures, symbiosis with other organisms, presence of contaminants, and these conditions can promote resistance to physical and chemical stressors that can, in turn, be used as ecotoxicological indicators [17]. For instance, the degradation of pollutants in the environment occurs by indigenous microorganisms that reduce their toxicity, their concentration and their toxic effect in the environment. Thus, they can potentially also serve as indicators of environmental pollution that generate a community response to pollutants [5].

Bioprospecting of yeasts in different habitats is a topic of great interest due to the derived industrial applications of their activities/properties [18,19,20]. Yeasts can also be used as model organisms to assess the toxicity and ecotoxicity of pesticides [21,22]. As notable examples, the yeast *Saccharomyces cerevisiae* has been studied as a model to assess the toxicity of contaminated soils and waters [23], as well as the degradation of organophosphorus compounds such as diazinon, using *Rhodotorula glutinis* and *R. rubra* [24], Malathi- on, Demeton-S and VX ps by *Saccharomyces cerevisiae* and Methamidophos by *S. rouxii* WY-3 [25,26].Plate diffusion and tube dilution techniques for MIC (Minimal Inhibitory Concentration) are commonly used for the evaluation of toxicity, with microorganisms as the probing target [27]. Bioassays with algae are assessed by agar diffusion tests [28], tests of diffusion with filter paper in agar [29] or agar diffusion assay (ADA) with different toxic concentrations [30]. There are also tests using halophilic microorganisms [31] such as those able to produce extracellular hydrolysates in agar with 10% of salt [32,33] and having developed screenings to detect esterase and lipase activity in agar [34], or by being able to turn agar opaque [35]. The effect of pesticides can also be measured with culture dependent and independent methods in soil bacterial communities [35] and in vitro techniques to measure esterase activities [36], among others.

Therefore, the aim of this study is to explore, retrieve and test, out of the yeast diversity that is harbored in the marine sediments from Cartagena Bay, Southern Caribbean Sea, yeast isolates that exhibit metabolic and response traits that could have the potential to serve as a biological model for microbial ecotoxicological evaluation using Chlorpyrifos as the environmental stressor.

## 2. Materials and Methods

### 2.1. Chemical Reagents

Chemical reagents used in this investigation are of analytical grade. These are: glucose, lactose, maltose, sodium chloride, ammonium nitrate, monopotassium phosphate, disodium sulfate, magnesium sulfate, calcium chloride, and zinc sulfate (Merck KGaA, Darmstadt, Germany), agarose, acrylamide, bis-acrylamide, urea, Tween 80, Chlorpyrifos (CP) 99.7% and 3,5,6-trichloro-2-pyridinol (TCP) 99.3% (Sigma-Aldrich Merck KGaA, Darmstadt, Germany).

### 2.2. Microbiological Culture Media

Agar, peptone, malt extract, and yeast extract (Becton Dickinson, BD and Company East Rutherford, Franklin Lakes, NJ, USA) were used. To prepare saline malt extract agar (20 g malt extract, 10 g NaCl, 17 g agar, 200 ug/L chloramphenicol and 1000 mL bidistilled water) were taken. For the PDA potato dextrose agar were (200 g of potato, 20 g of glucose, 15 g of agar and 1000 mL of bidistilled water). YPD broth was prepared using (10 g yeast extract, 20 g peptone, 20 g dextrose, 0.5 g chloramphenicol, and 1000 mL bidistilled water). To obtain solid media, 15 g/L of agar were added to the broths. The lactose broth was prepared with (meat extract 3 g, peptone 5 g, lactose 5 g, distilled water 1000 mL). The minimal salt medium, MSM was obtained with (NH_4_NO_3_ 12 g, KH_2_PO_4_ 8 g, Na_2_SO_4_ 2 g, KCl 4 g, MgSO_4.7_H_2_O 1 g, CaC_l2_ 0.5 g, ZnSO_4.7_H_2_O 0.26 g, NaCl 20 g, chloramphenicol 200 ug/L and bidistilled water 1000 mL).

### 2.3. Sampling and Preparation of Sediments

Samples of marine surface sediments were taken in Cartagena Bay, Colombia, in 9 stations: E1 (Latitude 10.390262/Longitude −75.533290), E2 (Latitude 10.3199 29/Longitude −75.541060), E3 (Latitude 10.318369/Longitude −75.526882), E4 (Latitude 10.325081/Longitude −75.517751), E5 (Latitude 10.36755/Longitude −75.510444), E6 (Latitude 10.375544/Longitude -75.516585), E7 (Latitude 10.382154 / Longitude −75.532465), E8 (Latitude 10.391596/Longitude −75.551964) y E9 (Latitude 10.397261/Longitude −75.540941), taking into account a distance of 300–500 m from sources of pollution.

Stations 1–3 in the Bay of Cartagena are influenced by the freshwater outlet of the Magdalena River, which collects its water contaminated with residuals and various pollutants (heavy metals, pesticides, hydrocarbons, among others); stations 4–5 with the Mamonal industrial zone (hydrocarbons, metals, pesticides, etc.); station 6 with Bosque industrial zone (old industrial zone in urban area) and station 7–9 by wastewater and urban rains (organic matter, pathogenic microorganisms, etc.).

The sediments were taken with a dredger, Ekman type. Fresh sediments (FS) were collected in heat-sterilized glass flasks and ice-refrigerated until laboratory storage at −20 °C until their respective analyses. Lyophilized sediments (LS) were prepared the same way as FS, but the flask was filled halfway (the lyophilized sediment is easy to handle, better conservation and stability of its chemical and biological components); the wide-mouth glass jar previously frozen was covered with aluminum foil and parafilm where small perforations were made. The flasks of all stations were freeze dried under temperature conditions at −50 °C and a pressure of 0.1 PSI (FreeZone 1 series Labconco) for 36–48 h to ensure complete lyophilization.

### 2.4. Extraction and Quantification of Total DNA in Sediments

Extraction of the total DNA in each of the sediments of the sampling site stations (fresh sediment 0.5 g and lyophilized sediment 0.25 g) was performed with the Power Soil, DNA Isolation KIT (MO BIO Laboratories Inc.) following the manufacturer’s instructions. DNA quantitation was carried out on a Fluorometer QUBIT (Invitrogen. Waltham, MA, USA), taking 2 μL of DNA, and following the manufacturer’s instructions, reporting the results in μg/mL. DNA samples were preserved at −20 °C [37,38,39].

### 2.5. DGGE (Denaturing Gradient Gel Electrophoresis) [38,39]

PCR amplification (Polymerase Chain Reaction): ITS4-EF4 primers (ITS4: sequence 5′-TCCTCCGCTTATTGATATGC-3′; EF4: sequence 5′-GGAAGGG[G/A]TGTATTTATTAG-3′), were used in a first reaction with a total volume of 25 μL (water 19.25 μL, tampon 2.5 μL, MgCl_2_ 1.25 μL, ITS4 0.5 μL, EF4 0.5 μL, dNTP 0.5 μL, BSA 1% 0.25 μL, Taq 0.25 μL, DNA 0.25 μL) and PCR conditions 94 °C for 5 min, 35 cycles: 94 °C for 30 s, 55 °C for 30 s, 72 ° C for 1.30 min, 72 °C for 5 min and finish at 4 °C. A second round ITS1FGC-ITS2 primers (IT1FGC: sequence 5′CGCCCGCCGCGCGCGGCGGCGGGGGCACGGGGGGCTTGGCATTTAGAGGAAGTBAA-3′; ITS2: sequence 5′-GCTGCGTTCTTCATCGATGC-3′) with total reaction volume 50 μL (water 39 μL, T10X 5 μL, ITS2 1 μL, ITS1FGC 1 μL, dNTP 1 μL, Form 0.5 μL, Taq 0.5 μL, DNA 2 μL) and PCR conditions 94 °C for 5 min, 35 cycles: 55 °C for 30 s, 72 °C for 30 s, 72 °C for 10 min. Amplicons were checked by agarose gel electrophoresis.

An 8% acrylamide gel with a denaturing gradient of 30–60% of formamide was prepared. Twenty μL of each sample amplicon were ran from each station in amplicon triplicate at 75 V for 16 h. The gel was colored for 1 h in SYBR green (Thermo Fisher Scientific, Waltham, MA, USA) solution. Resulting banding patterns in gels were scanned and analyzed using BioNumerics Software, v5.10 upplied by Applied Maths (Marcy-l’Étoile, Franceia).

### 2.6. Isolation and Phenotypic Characterization of Yeasts

Isolation and conservation. Yeasts were isolated from fresh sediment (SF) and lyophilized sediments (SL) samples; 1 g of sample were taken and mixed with a saline solution of 10 mL of 0.9% NaCl and 0.5 mL of Tween 20. Vortex was applied to the 1/10 dilution mixture for 2 min and standing for 2 min to decant particles. Then, 0.1 mL of dilutions 1/10 and 1/100 in saline solution were spread plated (triplicate) in malt extract agar (malt extract 2%) and incubated at 25 °C for 5–7 days. Each of the growths were spiked on malt extract agar for purification and each purified morphotype, seeded in duplicate in tubes with cotton in slants of potato dextrose agar, for storage at 4 °C (stocks of filamentous fungi and yeasts). In addition, YPD, Lactose and MSM broths (with and without chloramphenicol) were used for the isolation of more yeasts with Chlorpyrifos at 50 ppm, incubating YPD and Lactose at 25 °C for 5–7 days and shaking at 180 rpm the MSM for 30 days at room temperature (25–28 °C). The quality control was based on the maintenance of media, for a growth control and control of the sterilization [40,41].

Biochemical characterization of yeast. Purified colonies were plated on solid media and transferred to fresh media and cultures routinely checked microscopically for the presence of yeast morphologies and the absence of bacterial cells. The biochemical identification and assimilation of carbohydrates by yeasts was determined using the API 20 C AUX^®^ System (BioMérieux, Marcy-l’Étoile, France), where the growth of yeasts was observed by turbidity in each well with the carbohydrate, and compared with a negative control, following the manufacturer’s instructions [42]. Selected purified yeast strains were routinely maintained on YPD Agar 3% NaCl and incubated for 24 h at 25–28 °C; five colonies were transferred to YPD Broth 3% NaCl and incubated for 24 h at a temperature of 25–28 °C, at 0.5 of the MacFarland scale, obtaining the inoculum for subsequent experiments.

Symbiosis or antibiosis tests of yeasts isolated from sediments. With the purpose of using a mixture of yeasts or consortium for biodegradation processes, growth tests are made between them in agar, choosing those that do not produce inhibitory substances. Agar surface test: Each yeast was streaked out on the surface of YPD agar perpendicularly vs. the other yeasts in parallel, ensuring direct contact and incubated for 48 h at a temperature of 25–28 °C; the results were coded as symbiosis-growth (+), antibiosis-zone of inhibition of growth (−) and low antibiosis (o).

Agar surface tests for the detection of esterases/lipases in yeast. Two types of agar plates (Agar Tween 80-opacity and Agar Tween 80-indicator) were prepared, a yeast inoculum of 3 µL in triplicate and incubated for 5 days at a temperature of 25–28 °C. The test was considered positive for the Tween 80-opacity Agar when it was observed that a clearance halo around the inoculum emerged and a zone with color change from red to yellow. The halos were measured in millimeters (mm): 0 = Negative, 1–5 mm = Low, 6–10 mm = Medium and 11–15 mm = High.

Range of salt concentration and temperature growth. Yeast was inoculated in media with different concentrations of NaCl and temperatures: Agar YPD, YPD + 2% NaCl, YPD + 4% NaCl, YPD + 10% NaCl and YPD + 25% NaCl and incubated at 4 °C, 25 °C, 37 °C and 45 °C for 48 h. After that, the growth result was recorded.

### 2.7. Microbiological Test for Ecotoxicology

Pilot tests for the determination of Minimum Inhibitory Concentration (MIC) of Chlorpyrifos and its metabolite TCP on agar surface.

Pilot test 1: On the surface in YPD agar, 100 µL of yeast inoculum was spread homogenously with a sterile swab, and 6 mm paper disks were placed. Then, 25 µL of CP and TCP were added over the disks at concentrations of 500, 250, 125, 60, 30, 15, 0 ppm. After incubation for 48 h at a temperature of 25–28 °C, zones of inhibitions around the sensi-disks were registered.

Pilot test 2: On the surface in YPD Agar, 100 µL of a yeast was spread homogenously with a sterile swab, adding 5 µL of CP and TCP directly on the surface at concentrations of 500, 250, 125, 60, 30, 15, 3.7, 1.8, 0.9, 0.45, 0.23, 0.12, 0.06, 0.03, 0.0015 and, 0 ppm, incubating for 24 h at a temperature of 37 °C and seeing zone of growth inhibition (+); MIC is defined as the concentration where growth begins in the contact zone of the substance analyzed (−).

Final test for MIC determination: On the surface in YPD Agar 100 µL of four yeasts were homogenously with a sterile swab, adding 5 µL CP and TCP in triplicates directly on the surface at concentrations of 500, 250, 125, 60, 30, 15, 3.7, 1.8, 0.9, 0.45, 0.23, 0.12, 0.06, 0.03, 0.0015 and, 0 ppm, followed by incubation for 24 h at a temperature of 37 °C. Zone of growth inhibition were recorded (+); MIC was defined as the concentration where growth begins in the contact zone of the substance analyzed (−).

## 3. Results and Discussion

### 3.1. Yeast/fungal Diversity by Molecular Fingerprinting Profiling by DGGE (Denaturing Gradient Gel Electrophoresis) in DNA from Marine Sediments at Cartagena Bay

**Figure 1:**
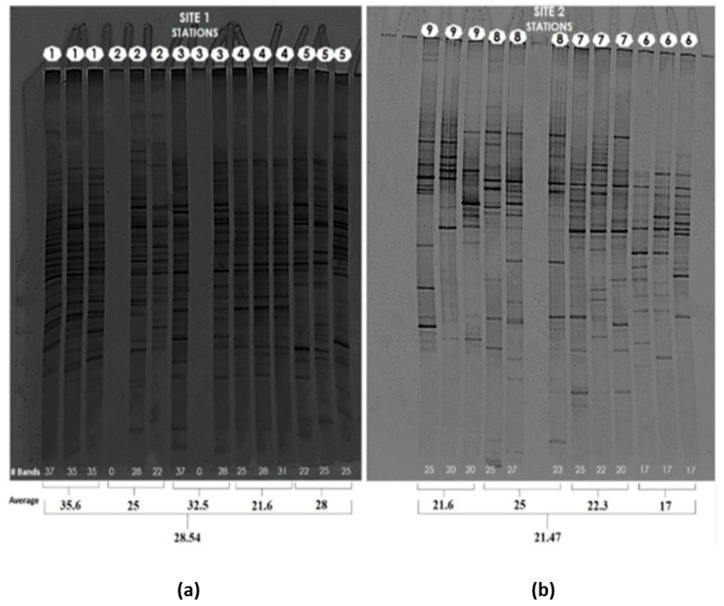

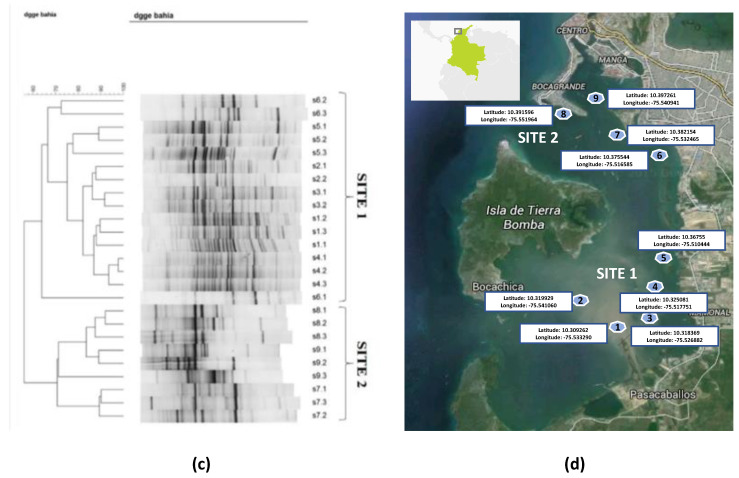
Acrylamide gel and dendrogram DGGE of sediments in Cartagena’s Bay. (**a**) Acrylamide gel in site 1; (**b**) Acrylamide gel in site 2; (**c**) Dendrogram DGGE site 1 and 2. (**d**) Study area.

DNA extraction from lyophilized sediments (LS) and fresh sediments (FS) was attained. Higher concentrations were generally obtained in the LS samples; therefore, LS extractions were used in molecular fingerprinting assays to determine global patterns of fungal diversity and similarity across the samples analyzed using DGGE technique targeting ITS fungal amplicons. The results showed the closer clustering/similarity of profiles according to the sampling station/site, indicating a consistent banding pattern obtained from the samples that are closer in location, and technical replicate, and the rich fungal diversity present in the marine sediments analyzed, where E1 to E5 (site 1) seems to be more diverse with respect to the E6 to E9 (site 2). The dendrogram can be seen in the two groups or sites, where site 1 has a greater diversity of fungi compared to site 2. It should be noted that station 6 of site 2 was incorporated into site 1, as this station is close to a traditional industrial area in the urban area (Bosque), where some industries: automotive workshops and other industrial activities and discharge, affect the Bay (Figure 1).

The technique of lyophilization for samples subjected to DGGE analyses [43] was used on marine sediments, allowing a better preservation of these types of environmental samples.

Stations 1–6 are grouped in site 1 and appear to have higher fungal diversity as assessed by this method. This could be explained by the higher input of domestic and industrial sewage contamination in these locations [44,45,46].

### 3.2. Isolation and Biochemical Characterization of Yeasts

Isolation on Agar Malt (MAE). Isolation of morphotypes from fresh sediments of each station was performed in triplicate and at 10^−1^ and 10^−2^ dilutions. After incubation for 72 h, microbial growth was observed in plates from E1 to E5 (site 1). From site 1, yeast strain G35 was isolated from station 1, and yeast strain G43 was isolated from station 4. They could represent different species given their contrasting origins and distinct diverse background of DGGE patterns.

Isolation of yeasts in broth YPD, lactose broth and MSM broth with 50 ppm of CP. One additional morphotype was obtained with YPD medium (YPD1) and eight more morphotypes could be isolated from lactose broth (LAC4, LAC6 and LAC7) and from MSM with CP (CP1, CP2, CP4 and CP8). 

Biochemical characterization of yeast. Yeasts isolated from surface sediments in the Bay of Cartagena were characterized biochemically with API 20 C. Their morphologies can be seen in Table 1.

Out of the total morphotypes isolated yeasts biochemically characterized, six were classified as belonging to the genus *Candida*, three of *Rhodotorula* and one of *Cryptococcus*. The genus *Candida* is recovered more often in the culture and media used for yeast isolation in these sediments, highlighting its isolation from the MSM medium with 50 ppm CP, as in MAE, while the *Rhodotorula* genus was isolated only from lactose broth. Marine yeasts can be saprophytic or pathogenic to plants or animals and are regularly found in aerobic marine ecosystems [47]. The first marine yeasts were described in samples from the Atlantic Ocean, and the most commonly isolated genera were *Candida, Cryptococcus, Debaryomyces* and *Rhodotorula*, in agreement with further studies [48,49]. In estuarine sediments in Brazil, the genus *Candida* was found as the most commonly recovered in culture-dependent studies [50,51], with *Candida tropicalis* and other animal pathogens prevailing, but not excluding their functions in open environments of decomposition, nutrient cycle and biotransformation of xenobiotic compounds in contaminated environments [52].

In Colombia, reports on the diversity of marine yeasts is rather limited. Sediments and water are important habitats in the search for species with industrial, biotechnological, and bioremediation potential. In two artificial lakes, the genera of *Candida, Cryptococcus, Williopsis, Hanseniaspora, Rhodotorula, Saccharomyces, Torulaspora, Tricosporum* and *Yarrowia* were reported in isolated yeasts [53].

Assimilation of carbohydrates by yeast isolated from sediments. Carbohydrates are a source of carbon and energy for yeasts, and thus have a metabolism that can be used by biotechnological processes. The assimilation of carbohydrates can determine metabolic versatility and can supply a more flexible medium to grow in ecotoxicological testing assays. When analyzing these carbohydrate assimilation responses by the marine yeast morphotypes isolated from the surface sediments, the general percentages of assimilation of each carbohydrate are obtained by all the yeasts studied.

**Figure 2:**
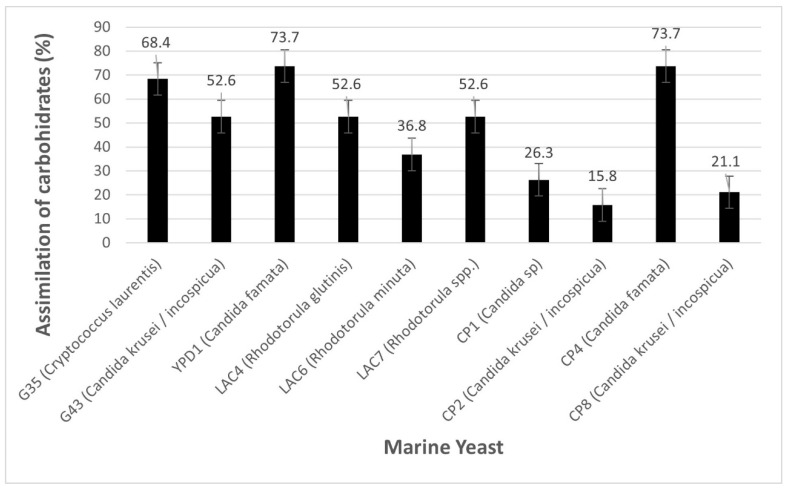
Percentage of assimilation of all carbohydrates by yeasts.

Glucose assimilation by yeasts is total (100%) with 90% glycerol and N-acetyl-glucosamine. The carbohydrate assimilation percentage of the yeasts isolated and studied is classified into four categories (0–25% = Low, 26–50% = Medium, 51–75% = Fairly High, 76–100% = High), where they were 16% high, 10% quite high, 58% medium and 16% low. Taking into account each yeast and seeing the percentage of assimilation of all carbohydrates, the strains that could be more versatile in the medium were highlighted and adapted in terms of the composition of their media, for cultivation and testing in the laboratory (Figure 2).

When seeing morphotypes of yeasts individually and by assimilation of carbohydrates in general, the highest category was Fairly High with 60% (morphs G35, G43, YPD1, LAC4, LAC7 and CP4), Medium with 20% (morphs LAC6 and CP1), Low with 20% (Morphs CP2 and CP8) and High = 0%; the versatility of the assimilation of carbohydrates of these isolated yeasts is highlighted.

The growth of yeasts in a habitat depends on the sugars and other nutrients that are available [54]. The assimilation of carbohydrates from yeasts has strengthened their identification due to the difference between different genera [55]. When comparing carbohydrate assimilation in this study with one *Rhodotorula glutinis* isolated from clinical and environmental settings, glucose assimilation was 100%, while glycerol assimilation in this study was 90%, compared to 50% [56].

### 3.3. Agar Surface Tests

Symbiosis /antibiosis tests of yeasts isolated from sediments. The analysis of symbiosis and antibiosis of isolated yeasts was tested to determine if the strains could potentially be used for microbial ecotoxicology testing or CP metabolization assays as a consortium. A low antibiosis was observed between the strain combinations assessed. Microbial consortia involving yeasts such as *Saccharomyces* with bacteria are of great importance for the improvement in the production of metabolites in biotechnological processes [57]. A consortium of yeast composed of *Saccharomyces* and *Candida* has been used to produce multiple enzymes [58]. A microalgae consortium with *Candida* yeast isolated from wastewater were tested to increase fatty acids in biodiesel production [59], as well as bioethanol production with a consortium of *Candida* and *Hanseniaspora* yeasts [60] and oil production with a consortium of the microalgae *Chlamydomonas* and the oil yeast *Lipomyces* [61].

Agar surface tests for the detection of esterases/lipases in yeast. In bioremediation processes, microorganisms are essential to degrade contaminants, involving enzymes for their degradation, and in the case of the organophosphate insecticide Chlorpyrifos, esterases/ lipases are involved. A pilot test was carried out using two markers (opacity and neutral red), where the opacity was ruled out due to the lack of overall clarity in the media, while the neutral red showed very clear color changes, the latter being the test chosen for the determination of lipase activity (Table 2).

A positive response to the enzyme was detected in all morphotypes of yeasts studied, distributed between medium and high response in 64% (CP2, YPD1, CP8, CP1, LAC7, G43, LAC6) and 36% (G35, LAC4, Control, CP4), respectively. There is a link between such degrading responses and the organophosphate Chlorpyrifos as detailed below:

Among the microorganisms that produce lipases and phospholipases such as filamentous fungi of the genera *Geotrichum, Aspergillus, Penicilllium* [62,63,64], there is potential biotechnological application given the biodegradation of Chlorpyrifos with phosphotriesterases [65]. The genus of yeast *Candida, Torulopsis, Issatchenkia, Yarrowia, Saccharomyces, Debaromyces, Cryptococcus, Hansenula, Pichia, Kluyveromyces, Wickerhamonyces, Metschnikowia, Rhodotorula, Trichosporum* have been found [64,66,67,68,69,70], as well as the yeast *Guehomyces* isolated in the Antarctic [70], such as the filamentous psychotropic fungi *Penicillium* and *Pseudogymnoascus* [71].

Yeast growth in different conditions of salt concentration and temperature. The different extreme abiotic factors that microorganisms can withstand are of great importance for their application in microbial biotechnology, so isolated yeasts are studied against growth at different temperatures and salt concentration. Tolerable and optimal salt concentrations and temperature were studied on our yeast collection (Table 3).

The growth of the yeasts on the agar surface was determined by visualizing many colonies, such as those obtained from temperatures of 25 °C and 37 °C (mesophilic), up to salt concentration of 4%, with some slowly growing at 10% (halotolerant). Growths at 4 °C sustained growth at 25% salt. Likewise, growth at 45 °C had better proliferation at salt concentrations up to 2% of salt, while not over 4%.

Of the extreme yeast genera, six were *Candida* and one was *Rhodotorula*. 

The reported diversity of yeasts in aquatic ecosystems showing flexibility to tolerate different ranges of salinity, temperature, oxygen saturation levels and different stressors in the environment, are from *Rhodotorula*, *Candida* and *Cryptococcus* genera. Xerotolerant or osmotolerant yeasts have been isolated at NaCl concentrations of 10 and 15%, and are usually isolates of *Candida, Rhodotorula, Torulopsys, Pichia* and *Debaryomyces* genera [72]. The isolated genera in this study *Candida* and *Rhodotorula*, which showed growth at temperatures of 4 and 45 °C, as well as NaCl concentrations of 2, 4, 10 and 25%, agree with the affiliation of strains and features reported in previous studies [73,74,75,76,77].

### 3.4. Microbiological Test with Application in Ecotoxicology

Pilot tests for the determination of MIC of Chlorpyrifos and its metabolite TCP on agar surface. The effect of different concentrations of the insecticide Chlorpyrifos and its TCP metabolite showed a very small zone of inhibition around the paper disc in study pilot 1, probably due to the low diffusion coefficient of the substances studied. Therefore, in pilot test 2, more dilutions of the substances were made, showing clear zones of inhibited growth on the amount of substance in different concentrations, having a clearer behavior than the previous technique, which allowed choosing it to test the final technique on the four yeasts strains selected.

**Figure 3:**
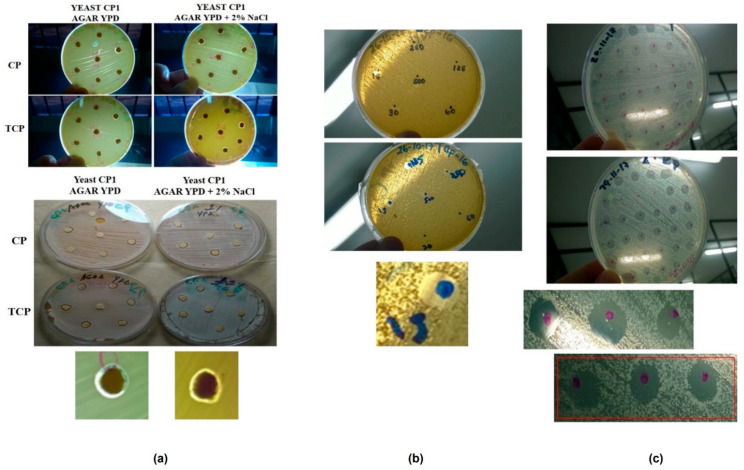
Inhibition of yeasts on agar surface. (**a**) Pilot test 1. Test of the use of sensidiscs on agar with concentrations of substance. (**b**) Pilot test 2. Substance concentration test directly on agar. (**c**) Definitive test for MIC. Determination with substance concentrations on agar.

Final test for MIC determination: As the pilot test 2 showed, consistent and clear results were obtained, where clear zones of inhibition and MIC values could be determined for each substance in each of the four yeast strains studied. (Figure 3).

MIC determination. The technique was tested with all the dilutions made with CP and TCP, to find the concentration that inhibited the yeast in the area of the drop volume placed on the previously diffused agar with the yeast to be studied (Table 4).

The minimum inhibitory concentration (CMI) of CP and TCP in the four selected yeast strains showed a higher TCP toxicity. Regarding the development of the microbial ecotoxicology test, the MICs found in these yeasts were between 0.45 and 1.8 ppm for CP and 0.45 ppm for TCP. 

## 4. Conclusions

We report here the characterization of the patterns of fungal diversity, yeast strain isolation, characterization and testing, aiming to stablish a microbial ecotoxicology test using Chlorpyrifos as a model environmental stressor, using marine sediment in a Southern Caribbean Bay (Cartagena) as the main source. Yeast showed assimilation of carbohydrates of glucose 100% and glycerol, *n*-acetylglucosamine by 90%. A low antibiosis was evidenced between isolates, thus allowing the possible use of a microbial consortium for the test. The presence of esterase/lipase was determined in all isolated yeasts. The growth of yeasts in extreme temperature conditions was demonstrated (4 °C/25 °C/37 °C/45 °C) and salt concentrations (without salt/2%/4% /10%/25%), finding extremophile yeasts. Therefore, a microbial ecotoxicology test developed with these strains could be compatible with the culturing of different carbon source compositions, samples of varying salt concentrations or different incubation temperatures. The minimum inhibitory concentration of CP and TCP in the four selected yeast strains showed a higher TCP toxicity. Regarding the development of the microbial ecotoxicology test, the MICs found in these yeasts were between 0.45 and 1.8 ppm for CP and 0.45 ppm for TCP, where strains 1 and 2 differentiated between these substances, with the TCP metabolite being the lower concentration, which it inhibited, being more toxic; however, for the other 2 strains, it did not differ at the lowest concentration of 0.45 ppm. Some authors have shown responses to CP in unicellular microorganisms such as *Aliivibrio fischeri* bacteria, with 2.84, 0.046 and 2.94 ppm [78,79,80]. Additionally, with the cianobacteria *Synechocystis* 0.074 ppm, and *Gloeocapsa* 0.080–0.3 ppm [81], as in green algae *Ankistrodesmus* 22.44 ppm [82]. According to data published by the author, with *Aliivibrio fischeri*, a response to CP of 4.18 ppm and TCP ppm was found; with *Pseudokirchneriella subcapitata*, the response to CP was 4.93 ppm and to TCP 0.294 ppm [83]. The literature mentions the use of in vitro techniques with bacteria, such as agar diffusion, where MIC is proposed as the gold standard for measuring the antimicrobial activities of natural substances [84]. Different antifungal substances were tested against the *Fusarium* filamentous fungus by measuring MIC [85]. *Talaromyces* yeast was tested with a Candida control to determine the MIC in Petri dishes and to determine the response to antifungal substances, reaffirming its use as a gold standard test [86]. MIC was determined on agar with *Saccharomyces, Candida* and *Yarrowia* yeasts to determine the toxic response to chromium and manganese metals [87]. The data presented in this study are a first approach that implies deeper study with more appropriate techniques such as fluorometry, by which the effects of these substances can be measured in standard methods with the mentioned microorganisms, including yeasts, as a model.

## Figures and Tables

**Table 1:** Yeasts characterized biochemically isolated from bay sediments.

Yeast	API 20 C
**G35**	*Cryptococcus laurentis*
**G43**	*Candida spherica*
**YPD1**	*Candida famata*
**LAC4**	*Rhodotorula glutinis*
**LAC6**	*Rhodotorula minuta*
**LAC7**	*Rhodotorula* sp.
**CP1**	*Candida* sp.
**CP2**	*Candida krusei/inconspicua*
**CP4**	*Candida famate*
**CP8**	*Candida krusei/inconspicua*

**Table 2:** Lipase test on agar surface with neutral red.

Yeast Code	Yeast	Total Diameter (Average in mm)	SCALE (mm) Negative = 0; Low = 0.1–5; Medium = 5.1–10; High = 10.1–15
**G35**	*Cryptococcus laurentis*	10.3	High
**CP2**	*Candida krusei/inconspicua*	9	Medium
**YPD1**	*Candida famata*	5.3	Medium
**LAC4**	*Rhodotorula glutinis*	10.6	High
**C**	*Saccharomyces cereviciae*	10.6	High
**CP8**	*Candida krusei/inconspicua*	8	Medium
**CP1**	*Candida* sp.	8.3	Medium
**LAC7**	*Rhodotorula* sp.	10	Medium
**G43**	*Candida spherica*	10	Medium
**CP4**	*Candida famata*	12	High
**LAC6**	*Rhodotorula minuta*	10	Medium

**Table 3:** Extremophile yeasts found in marine sediments (concentration of salt and temperature). (X) Highlights the growth of yeasts at the temperatures and salt concentrations studied.

Extreme Yeast	Growth 4 °C	Growth 45 °C	Growth 2% NaCl	Growth 4% NaCl	Growth 10% NaCl	Growth 25% NaCl
**Control 51669** ***Saccharomyces cerevisiae***					X	
**43** ***Candida spherica***	**X**				**X**	**X**
**YPD1** ***Candida famata***		**X**		**X**	**X**	
**LAC6** ***Rhodotorula minuta***	**X**	**X**	**X**			
**CP1** ***Candida* sp.**		**X**		**X**		
**CP2** ***Candida krusei/incospicua***		**X**	**X**			
**CP8** ***Candida krusei/incospicua***		**X**		**X**		

**Table 4:** MIC with direct CP and TCP technique on agar surface (4 yeasts). (+) Yeast growth on agar. (-) No growth of yeast on the agar.

	7.5 ppm	3.7 ppm	1.8 ppm	0.9 ppm	0.45 ppm	0.23 ppm	0.12 ppm	0.06 ppm	0.03 ppm	0.015 ppm	C-	CMI ppm
**CP1**	**-**	**-**	**-**	**+**	**+**	**+**	**+**	**+**	**+**	**+**	**+**	**1.8**
**TCP1**	**-**	**-**	**-**	**-**	**-**	**+**	**+**	**+**	**+**	**+**	**+**	**0.45**
**CP2**	**-**	**-**	**-**	**-**	**+**	**+**	**+**	**+**	**+**	**+**	**+**	**0.9**
**TCP2**	**-**	**-**	**-**	**-**	**-**	**+**	**+**	**+**	**+**	**+**	**+**	**0.45**
**CP3**	**-**	**-**	**-**	**-**	**-**	**+**	**+**	**+**	**+**	**+**	**+**	**0.45**
**TCP3**	**-**	**-**	**-**	**-**	**-**	**+**	**+**	**+**	**+**	**+**	**+**	**0.45**
**CP4**	**-**	**-**	**-**	**-**	**-**	**+**	**+**	**+**	**+**	**+**	**+**	**0.45**
**TCP4**	**-**	**-**	**-**	**-**	**-**	**+**	**+**	**+**	**+**	**+**	**+**	**0.45**

## Data Availability

Not applicable.
